# Supplementary material for: Exploring the Structure and Chemistry of One-Dimensional and Two-Dimensional Lepidocrocite TiO2 at Atomic Resolution
Source: J Am Chem Soc. 2026 Jun 23;148(26):27562–9. doi: 10.1021/jacs.6c06220 (PMC13352613; doi:10.1021/jacs.6c06220)
Supplement: Supplementary file 1 [file ja6c06220_si_001.pdf]

## SUPPORTING INFORMATION

# Exploring the Structure and Chemistry of 1-Dimensional and 2- Dimensional Lepidocrocite $TiO_2$ at Atomic Resolution

*Eric Nestor Tseng,[a] Jonas Björk,[b,c] Risha Achaiah Iythichanda,[a,c] Wei Zheng,[b] Jie Zhou,[b] Hatim Alnoor,[a] Wei Hsiang Huang,[d,e] Ming-Hsien Lin,[f] Johanna Rosen,[b,c] Per O.Å. Persson.[a,c]**

[a] Thin Film Physics Division, Department of Physics, Chemistry and Biology (IFM), Linköping University, 58183 Linköping, Sweden

[b] Materials Design Division, Department of Physics, Chemistry and Biology (IFM), Linköping University, 58183 Linköping, Sweden

[c] Wallenberg Initiative Materials Science for Sustainability (WISE), Linköping University, Department of Physics, Chemistry and Biology (IFM), 58183 Linköping, Sweden

[d] National Synchrotron Radiation Research Center (NSRRC), Hsinchu City, 30076, Taiwan

[e] Sustainable Electrochemical Energy Development (SEED) Center, National Taiwan University of Science and Technology, Taipei, 106, Taiwan

[f] Department of Chemical and Materials Engineering, Chung Cheng Institute of Technology, National Defense University, Taoyuan, 335, Taiwan

*Corresponding author: per.persson@liu.se

KEYWORDS: Titania, 1D materials 2D materials, HRSTEM

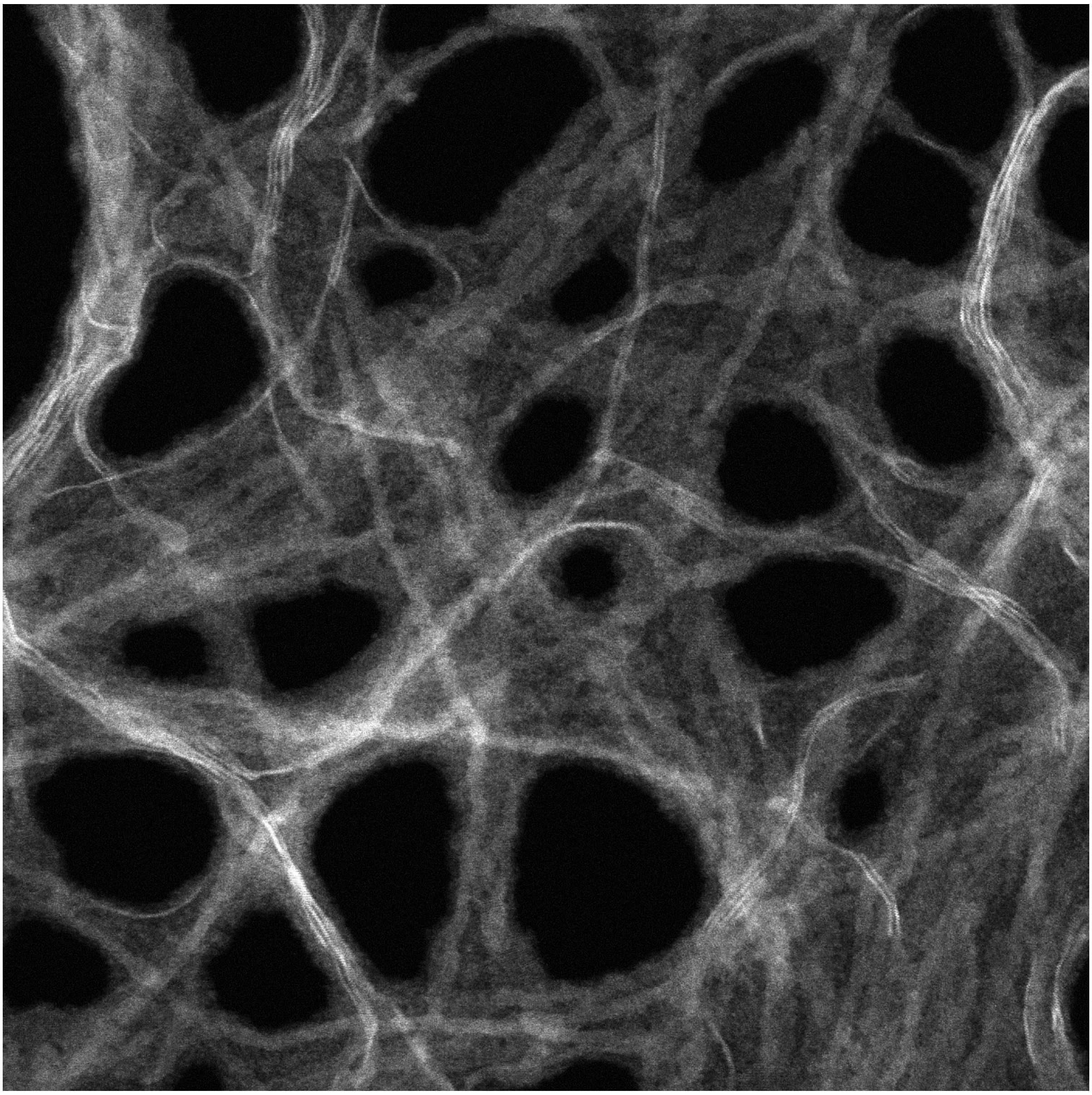

***Figure S1**: Overview image of the 1D filaments. Since the filaments are bending freely (although a preference for orienting themselves in the plane of the lacey carbon grid) it can be seen that filaments bend to orient themselves in cross section with respect to the electron beam. These cross-sectionally oriented filaments are seen as bright lines because of the increased thickness in projection. Locally, it is possible to observe single, double, triple, and multiple filaments. These are apparently crystalline and correspond to a unit cell thick lepidocrocite structure.*

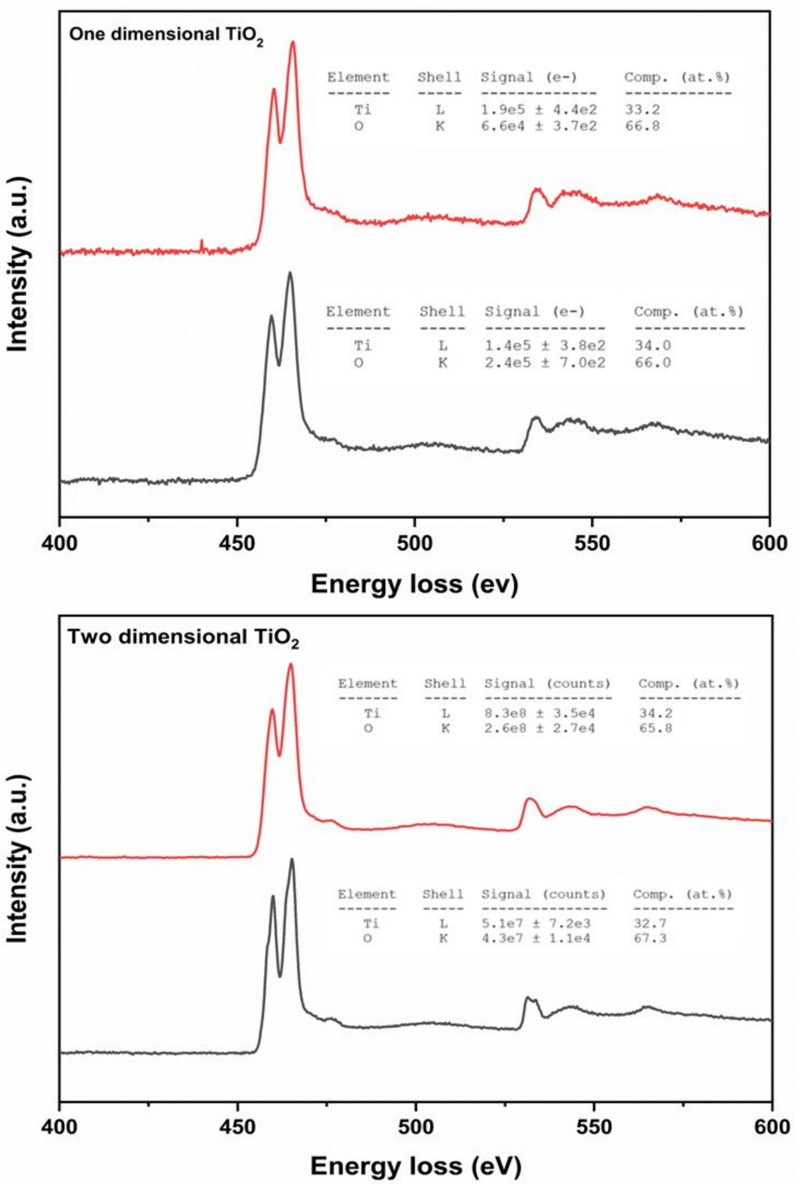


***Figure S2:*** *Electron energy loss spectra for one- and two-dimensional material with quantification results (inset)*

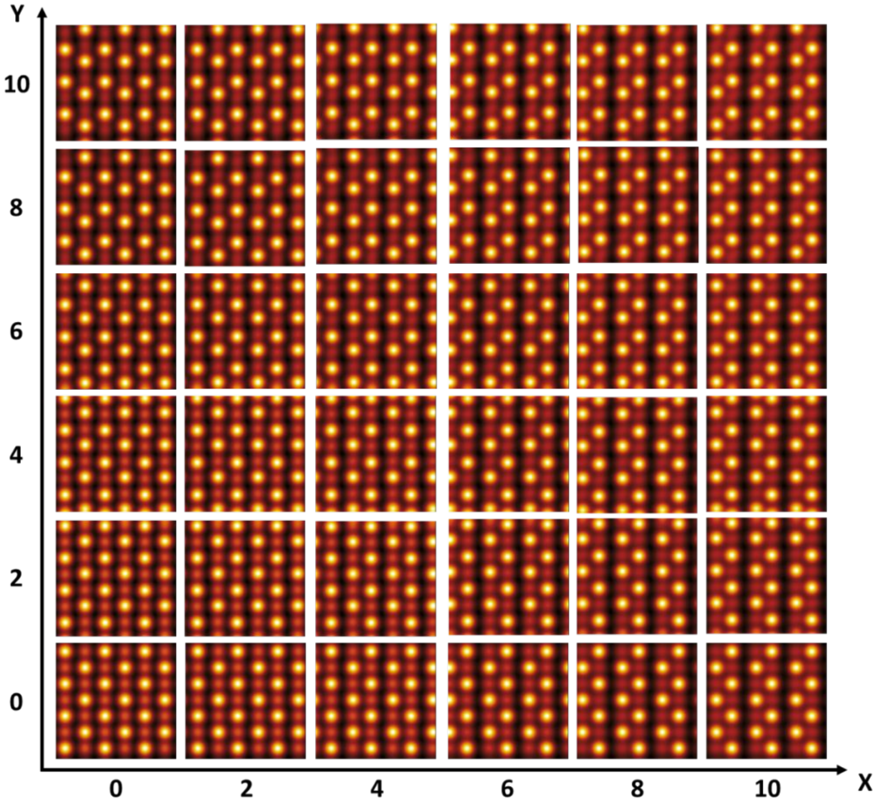


***Figure S3****: Simulated plan-view images of a single unit cell thick lepidocrocite TiO2 for a range of applied tilt in x & y directions (deg.).*

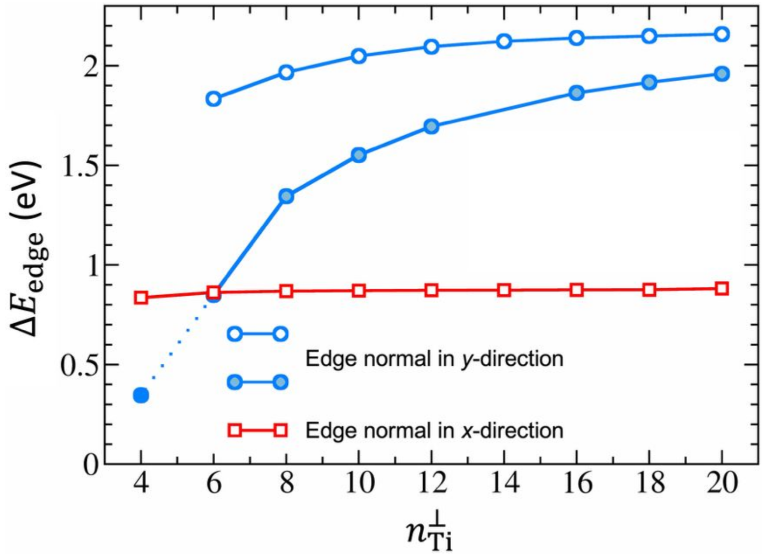


***Figure S4:*** *Comparison of the edge energies for TiO2 foils of different widths, with the edges oriented normal to the y- or x-directions. For edges normal to the y-axis, an edge configuration involving a structural reconstruction (filled circles) was found to be more stable than the unreconstructed configuration (empty circles). These reconstructed y-direction edges were considered when evaluating the effect of carbon on the edge energies in Fig. 5 of the main manuscript.*

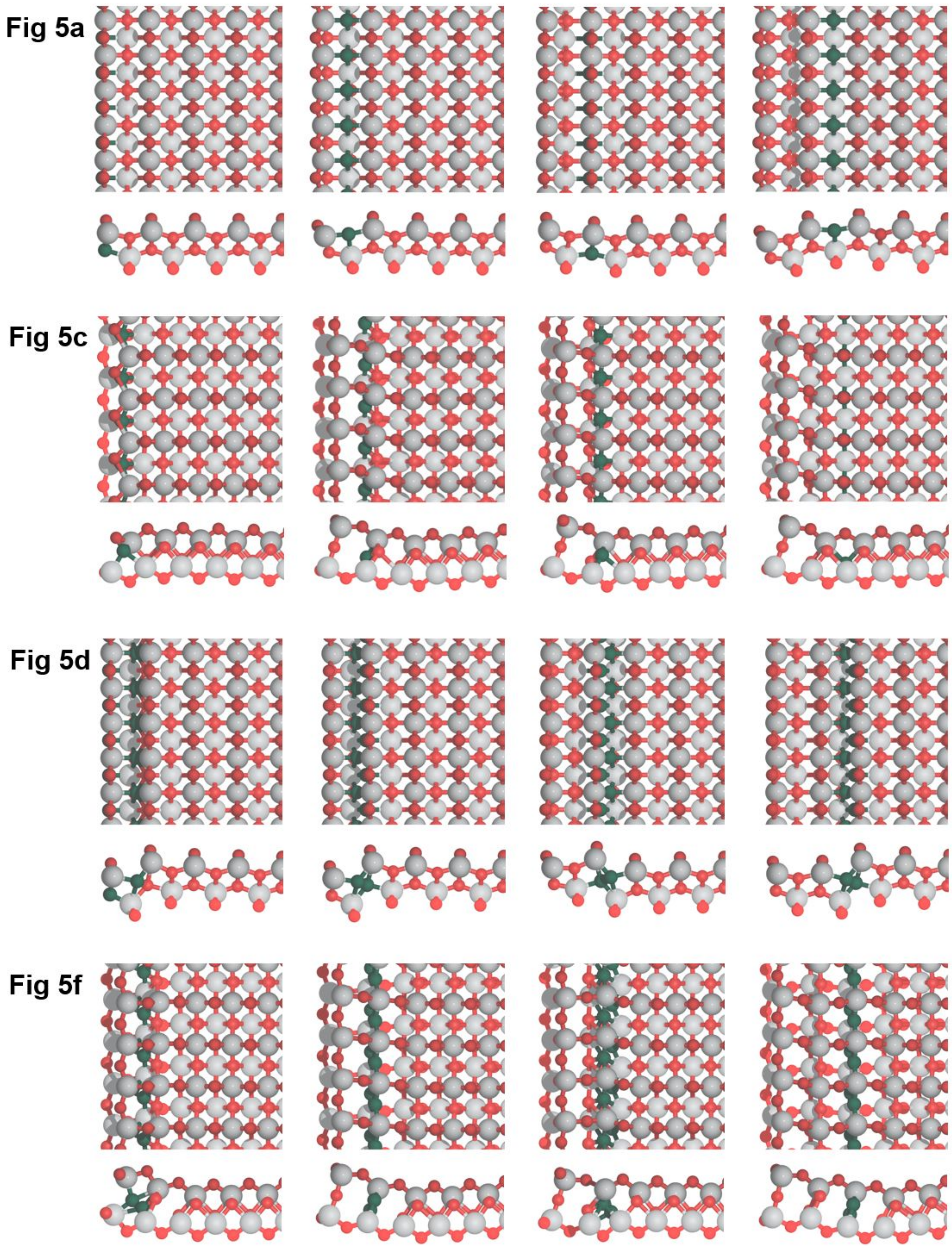


***Figure S5:*** *Higher magnification image of same structures as shown in figure 5.*

| Sample | Shell | CN | R(Å) | Debye-Waller factor ($\Delta\sigma^2$) | R-factor |
|---|---|---|---|---|---|
| $TiO_2$ | Ti - O | 4.25 ±0.850 | 1.83 ±0.0183 | 0.027 ±0.0054 | 3.8 x $10^{-2}$ |
| | Ti - Ti | 3.80 ±0.760 | 3.23 ±0.0323 | 0.130 ±0.0260 | |

***Table S1**: Coordination number (CN), bond distance (R), the Debye-Waller factor ($\Delta\sigma^2$) information from the fittingresults of the absolute Fourier transforms of extended fine structure region (k space) for two-dimensional $TiO_2$*
